# The paradigm shifts of periodontal regeneration strategy: From reparative manipulation to developmental engineering

**DOI:** 10.1016/j.bioactmat.2025.03.009

**Published:** 2025-03-18

**Authors:** Guanqi Liu, Junlong Xue, Xuan Zhou, Mixiao Gui, Ruidi Xia, Yanshu Zhang, Yihua Cai, Shuhua Li, Songtao Shi, Xueli Mao, Zetao Chen

**Affiliations:** a Hospital of Stomatology, Guanghua School of Stomatology, Sun Yat-sen University, and Guangdong Provincial Key Laboratory of Stomatology, Guangzhou, 510055, China; b Guangdong Research Center for Dental and Cranial Rehabilitation and Material Engineering, Guangzhou, 510055, China; c South China Center of Craniofacial Stem Cell Research, Guangzhou, 510055, China

**Keywords:** Periodontal regeneration, Developmental engineering, Immune microenvironment, Biomaterials

## Abstract

Ideal periodontal regeneration requires the integration of alveolar bone, periodontal ligament, and cementum, along with Sharpey's fibers for occlusal force resistance. However, physiological regeneration remains rare due to its intricate structure, making clinical regeneration a challenge. Periodontal ligament stem cells (PDLSCs), first isolated in 2004, hold the key to multi-directional differentiation into cementoblasts, fibroblasts, and osteoblasts. While traditional therapies like guided tissue regeneration (GTR) aim to activate PDLSCs, clinical outcomes are inconsistent, suggesting the need for additional strategies to enhance PDLSCs' functions. Advancements in molecular biotechnology have introduced the use of recombinant growth factors for tissue regeneration. However, maintaining their efficacy requires high doses, posing cost and safety issues. Multi-layered scaffolds combined with cell sheet technology offer new insights, but face production, ethical, and survival challenges. Immune regulation plays a crucial role in PDLSC-mediated regeneration. The concept of “coagulo-immunomodulation” has emerged, emphasizing the coupling of blood coagulation and immune responses for periodontal regeneration. Despite its potential, the clinical translation of immune-based strategies remains elusive. The “developmental engineering” approach, which mimics developmental events using embryonic-stage cells and microenvironments, shows promise. Our research group has made initial strides, indicating its potential as a viable solution for periodontal complex regeneration. However, further clinical trials and considerations are needed for successful clinical application. This review aims to summarize the strategic transitions in the development of periodontal regenerative materials and to propose prospective avenues for future development.

## Highlights

• The strategic shifts in periodontal regeneration are comprehensively reviewed, highlighting the limitations of current approaches and the necessity for more effective solutions.
•The innovative concept of “Developmental Engineering” is introduced, which aims to offer a more holistic and effective solution for regenerating the entire periodontal complex, including the periodontal ligament, alveolar bone, and cementum.
•The application of developmental engineering is discussed, emphasizing the combination of multidisciplinary approaches, including developmental biology, materials science, and engineering.

## Graphical abstract

**Figure:**
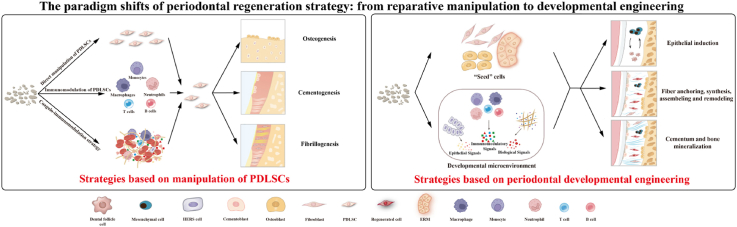

## 1 Introduction

The periodontium is an intricate and complex organ with interconnected soft and mineralized tissue, including epithelial tissue, gingival connective tissue, periodontal ligament, cementum and alveolar bone. Periodontal tissue defect is common in clinical practice, leading to tooth mobility, loss, and a myriad of associated clinical complications [1]. Ideal periodontal complex regeneration requires regeneration and integration of alveolar bone, periodontal ligament and cementum, which demands the newly formed Sharpey's fibers inserted into alveolar bone and cementum to withstand occlusal force. However, real periodontal complex regeneration is rarely observed under physiological conditions due to its complicated structure. Despite much significant efforts, achieving real periodontal regeneration in clinical practice remains a formidable challenge [2].

Periodontal ligament stem cells (PDLSCs) were first isolated and named by our team in 2004, which possesses the potentials to differentiate into the cementoblast, fibroblast and osteoblast [3]. PDLSCs are considered as the key effector cells for periodontal complex regeneration due to their multi-directional differentiation potentials. The biological principle of traditional periodontal regeneration therapy is based on activating the functional bioactivity of PDLSCs. Typically, guided tissue regeneration (GTR) employs barrier membranes as physical barriers to prevent the migration of the epithelial cells and gingival connective cells, inducing the initial attachment of PDLSCs to nude dentin and promoting periodontal complex regeneration. Although evidence indicates that GTR achieved successful clinical outcomes, in some clinical cases (such as intrabony defects and Class II mandibular furcation) the variability in clinical outcomes has been observed [4,5]. This suggests that apart from achieving preferential occupation of periodontal ligament tissue, other strategies such as promoting the PDLSCs differentiation, proliferation and mineralization are also required.

With the development of molecular biotechnology, the large-scale production of recombinant growth factors has led to the gradual discovery and application of the promoting effects in tissue regeneration, However, to maintain the long-term efficacy of growth factors locally, high doses are often required, which not only incurs high costs but also increases the risk of side effects. Although research has been conducted to develop carrier systems for the controlled and sustained release of growth factors, PDLSCs-mediated regeneration of multiple periodontal tissues is influenced by the comprehensive regulation of multiple factors. As such, a single growth factor is difficult to coordinate the regeneration of multiple periodontal tissues. Although delivery through carrier systems can solve the problem of long-term efficacy of factors to a certain extent, delivering multiple complex factors poses great difficulties in the design and synthesis of carrier systems. On the other hand, the simple combination of growth factors does not always achieve the expected outcomes [6]. Therefore, more precise regulation of PDLSCs in both time and space is needed to better achieve the precise assembly of various components of the periodontium, ultimately leading to the desired regenerative effect.

The multi-layered scaffold combined with cell sheet technology has emerged. Australian scholars Saso Ivanoski et al. designed a bilayer scaffold material composed of a periodontal ligament scaffold and an alveolar bone scaffold. The alveolar bone scaffold is made of β-tricalcium phosphate (β-TCP) and polycaprolactone (PCL), while the periodontal ligament scaffold is composed of electrospun membranes. Osteoblasts are implanted into the alveolar bone scaffold, and periodontal ligament cells are implanted into the periodontal ligament scaffold. When this cell-bilayer scaffold complex was implanted subcutaneously in nude mice, the formation of a structure comprising alveolar bone-periodontal ligament-cementum was finally observed [7]. In China, Chen's team used hydroxyapatite/tricalcium phosphate as a bone regeneration scaffold, and naturally isolated dentin as a cementum regeneration scaffold. Platelet-rich fibrin (PRF) was used as the periodontal ligament scaffold between the two, while bone marrow mesenchymal stem cells and periodontal ligament stem cell sheets were layered and combined [8]. In animal experiments, periodontal composite structures were also regenerated. The attempt to layer and combine multiple stem cell sheets provides new ideas for the regeneration of complex periodontal tissues. Although the multi-layered cell sheet technology has initially achieved the regeneration of multiple periodontal tissues, it is hindered by challenges such as complex production, difficulty in operation, high costs, and ethical and safety concerns. At the same time, during the *in vitro* culture of multi-layered scaffold-stem cell complex, the diffusion of nutrients and metabolic waste through each layer of the structure is limited, leading to the difficulty of survival of the implanted seed cells. Research by Liu et al. has shown that exogenous PDLSCs implanted into periodontal defects can promote the regeneration of periodontal tissues by inducing the polarization phenotype shift of macrophages [9], highlighting the important role of immune regulation in stem cell-mediated periodontal regeneration. The role of immune microenvironment regulation in PDLSCs-mediated periodontal regeneration has been gradually emphasized, and the development of periodontal regeneration materials targeting immune microenvironment regulation has become a hot spot. For example, materials designed to target polarization shift of macrophages have been explored. However, in the actual *in vivo* periodontal regeneration process, the composition and function of the immune microenvironment undergo sequential changes. As a result, the effect of promoting periodontal regeneration by immune regulation *in vivo* is often limited and less pronounced.

Our research group, after reviewing the immunopathophysiological processes of periodontal regeneration, has identified the initiation of blood coagulation and inflammation as the most critical events in the early stages of periodontal tissue regeneration [10]. These two processes share numerous immune cells and cytokines and exhibit mutual regulation. The blood clot contains a variety of immune cell components that participate in the immune regulation of PDLSC-mediated regeneration [11,12]. Therefore, we propose the concept of “coagulation-immunomodulation”, which involves coupling the regulation of coagulation and immune responses to promote periodontal regeneration *in vivo*. This strategy forms the basis for developing a new generation of periodontal regeneration materials. The introduction of this concept provides more specific and feasible application methods for strategies targeting immune microenvironment regulation for periodontal regeneration. While these strategies have shown some therapeutic effects in periodontal regeneration, most studies have struggled with clinical translation, often yielding ambiguous results *in vivo* and stalling at the preclinical stage [[13], [14], [15]]. Concurrently, our clinical research trials have found that autologous PDLSCs transplantation did not achieve significant clinical effects. These results suggest that periodontal regeneration strategies based solely on PDLSCs regulation are insufficient. To achieve successful periodontal complex regeneration, our understanding of the underlying biological processes must be updated.

With the evolution of regenerative medicine, the advanced strategy of “developmental engineering” has gained recognition and appreciation. The concept of “developmental engineering” refers to the promotion of tissue regeneration by recapitulating developmental events through the provision of embryonic-stage cells and the simulation of a developmental microenvironment during the tissue repair process [16]. However, the feasibility of this concept requires further in-depth research to provide evidence. We first need to elucidate the molecular regulatory mechanisms of periodontal development, as well as the similarities and differences between the periodontal development process and the periodontal defect repair process at the molecular level. Based on these biological foundations, corresponding materials should be developed to guide periodontal regeneration through developmental tissue engineering. Our research group has made preliminary attempts in this area and has achieved certain promising results. This suggests that developmental engineering holds great potential as a viable approach for achieving periodontal complex regeneration. However, further comprehensive clinical research trials are needed for true clinical translation, and more in-depth considerations, including cost estimation, potential ethical issues, and clinical outcomes, must be addressed.

## 2 Biological basis of periodontal multi-tissue regeneration

The periodontal multi-tissue repair and regeneration process can be divided into four overlapping phases: hemostasis, inflammation, tissue repair and wound remodeling. Following periodontal injury, hemostasis is initiated, leading to blood clot formation. Soluble fibrinogen is converted into insoluble fibrin to form a cross-linked fibrin network. The fibrin network not only serves as a temporary extracellular matrix for cell adhesion, proliferation and differentiation, but also regulates the activation of immune cascade, further regulating the immune microenvironment [17,18]. The immune microenvironment further regulates the biological behavior of various repair cells, promoting cells migration, proliferation and differentiation, which ultimately determines the outcome of periodontal regeneration. During this period, neighboring cells such as osteoblasts, fibroblasts, endothelial cells, and PDLSCs migrate into the defect area and commence the secretion of extracellular matrix collagen, thereby initiating the formation of an early repair matrix. Subsequently, the early repair matrix undergoes maturation and reconstruction, leading to the establishment of more organized periodontal tissue.

**Fig. 1:**
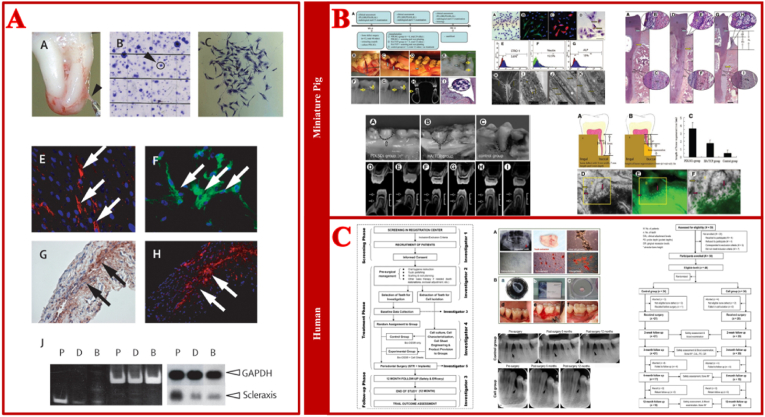
**Isolation, characterization and application of PDLSCs** [3,24,25]. (A) Investigation of multipotent postnatal stem cells from human periodontal ligament. Immunocytochemical staining showed that cultured PDLSCs were expressed STRO-1 and CD146/MUC18, two early mesenchymal progenitor markers. Immunohistochemical and fluorescence staining showed PDL tissue was positive for STRO-1 antibody. (B) PDLSCs-mediated treatment for periodontitis in miniature swine. (C) Randomized clinical trial of treatment of periodontal intrabony defects using autologous PDLSCs. The inlaid labels corresponds to the original figure's labels.

Among all repair cells involved in periodontal tissue regeneration, PDLSCs are the most important and promising cell for periodontal multi-tissue regeneration. First isolated and characterized by our team in 2004, PDLSCs possess multipotent self-renewal capacity [3]. PDLSCs highly express mesenchymal stem cell surface markers, such as STRO-1, CD146/MUC18, and have demonstrated significant potential of multidirectional differentiation, promoting periodontal tissue regeneration both *in vitro* and *in vivo*. PDLSCs are able to synthesize collagen fibers, form alveolar bone and cementum under specific induction [19]. Studies have demonstrated that PDLSCs also exhibit high expression of mesenchymal stem cell surface markers, including CD29, CD44, CD105, and STRO-1 [3,20]. PDLSCs have demonstrated remarkable potential in craniofacial tissue engineering (TE), including periodontal TE and cranial TE. For example, PDLSCs cell sheets combined with Bio-Gide® collagen membrane could obviously enhance alveolar bone volume and promote the formation of cementum-like structures and fibers in a rat periodontal defect model [9]. Another study showed that allogeneic PDLSCs seeded onto Gelfoam® had a marked ability to repair periodontal defects by regenerating bone, PDL and cementum-like tissue *in vivo* [21]. In cranial TE, a biomimetic construct composed of hydroxyapatite modified with an in vitro-derived extracellular matrix (HA-ECM) and seeded with PDLSCs has shown excellent repair of cranial bone defects in a rat model [22]. Furthermore, PDLSCs combined with a nanohydroxyapatite-coated genipin-chitosan conjunction scaffold (HGCCS) has shown promising outcomes of cranial bone repair [23]. Our research team has applied PDLSCs in treating periodontal defects in a porcine model of periodontitis and achieved favorable periodontal regeneration effect. This finding reveals the critical role of PDLSCs in periodontal regeneration [24]. However, in 2016, we conducted a single-center, randomized trial utilizing autologous periodontal ligament stem cells (PDLSCs) in conjunction with bovine-derived bone mineral materials to address periodontal intrabony defects. Patients enrolled in the study were randomly assigned to either the Cell group, which received treatment with guided tissue regeneration (GTR) and PDLSC sheets combined with Bio-oss (®), or the Control group, which received GTR and Bio-oss (®) without the inclusion of stem cells. However, no statistically significant differences were observed between the Cell group and the Control group on periodontal regeneration effect [25]. This suggests that the microenvironment in which PDLSCs are situated has a significant impact on their ability to facilitate multi-tissue regeneration in clinical periodontal applications. The multipotent differentiation capabilities of PDLSCs must be activated and orderly regulated within the *in vivo* microenvironment to effectively achieve functional periodontal regeneration (Fig. 1).

## 3 Paradigm shifts of periodontal regeneration strategies

### 3.1 Periodontal regeneration strategy based on direct manipulation of PDLSCs

Bone substitutes are widely used in clinical practice, which could provide space and promote alveolar bone regeneration. The physical structure of bone substitute was similar to natural bone, which could serve as a scaffold for cell migration and facilitating osteoblast differentiation and bone formation. Compared to using a barrier membrane alone, the combination with bone substitutes could improve the clinical outcome of alveolar bone regeneration [26,27]. However, this approach does not improve the periodontal complex regeneration. Bone substitutes are derived from bone and may not be specifically tailored for periodontal regeneration. Given the substantial differences in the biological processes underlying bone regeneration and periodontal complex regeneration—including variations in tissue structure, the types of repair cells involved, and the mechanisms of repair—it is essential to further modify bone substitutes to better meet the specific requirements of periodontal regeneration.

With the growing recognition of the pivotal role of periodontal ligament stem cells (PDLSCs) in periodontal complex regeneration over the past few decades, initial modification strategies have focused on enhancing the multi-directional differentiation potential of PDLSCs. Since multi-directional differentiation of PDLSCs is crucial for periodontal ligament complex regeneration, extensive efforts have been dedicated to promote the multi-directional differentiation of PDLSCs [[28], [29], [30]].

**Fig. 2:**
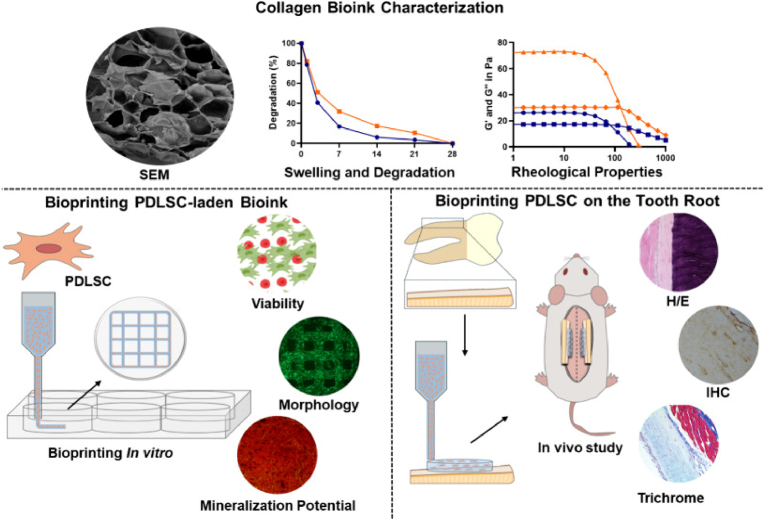
**Periodontal regeneration strategy based on direct manipulation of PDLSCs** [36]. A collagen-based bioink mimicking the native extracellular matrix conditions and carrying PDLSCs was constructed. This bioprinting scaffolds direct manipulated PDLSCs and had great potential for PDL regeneration as the scaffolds preserve cell viability, stimulate PDLSCs differentiation, demonstrate biocompatibility and suitable PDLSCs attachment to the root surface both *in vitro* and *in vivo*.

The function of PDLSCs could be enhanced by modulating the topography of biomaterials [31,32]. For example, Mao et al. fabricated hydroxyapatite (HA) bioceramics with a micro-nano-hybrid surface (mnHA), combining nanorods and microrods. This surface modification enhanced the attachment, spreading, proliferation, alkaline phosphatase (ALP) activity, and cementogenic differentiation of PDLSCs by activating Wnt signaling pathway [33]. Daghrery et al. applied nanostructured fluorinated calcium phosphate coatings on polycaprolactone (PCL) scaffolds. This modification upregulated osteogenic genes such as COL1A, OCN, and RUNX2 in PDLSCs [34]. In a study by Thattaruparambil et al. PDLSCs were bioprinted in gelatin methacryloyl (GelMA) hydrogels at varying concentrations. The high-concentration GelMA hydrogel provided a stiffer environment, which influenced the ephrinB2/EphB4 signaling pathway, leading to increased osteogenic differentiation [35]. Another research by Souza et al. constructed a bioprinted PDLSC-laden collagen scaffold, which exhibits great potential for PDL regeneration as the scaffolds preserve cell viability, stimulate PDLSCs differentiation, and demonstrate biocompatibility. The scaffold could be designed to target areas involving different PDL fibers’ organization through the evidence of the shear forces of bioprinting influencing cell alignment [36] (Fig. 2). While these approaches have shown success in preclinical studies, scaling them up for clinical applications remains a challenge. These methods offer limited regulatory effect on stem cells and involve intricate and complex fabrication processes. The high operational difficulty, expensive costs, and potential ethical and safety concerns further hinder their clinical application.

On the other hand, in order to more precisely stimulate specific aspects of PDLSCs capacity, bioactive factors such as recombinant cytokines and metal ions are used for biomaterials modification. Topical application of recombinant cytokines is an effective method to stimulate endogenous PDLSCs. For instance, Takeda et al. used brain-derived neurotrophic factor (BDNF) in dogs periodontal defects, which stimulated the formation of new alveolar bone, cementum and connective new fibers inserted into the newly formed cementum and bone [37]. Moreover, Kinoshita et al. utilized recombinant human bone morphogenetic protein-2 (rhBMP-2) to horizontal circumferential defects in beagle dogs, which showed new cementum with Sharpey's fibers on the instrumented root surface [38]. Similarly, the soluble cytokine IL-33 was used to promote PDLSCs' capacity. Research showed that IL-33 could increase PDLSCs' clonogenicity, proliferation, and expression of pluripotency markers, improving the osteogenesis of PDLSCs [39]. The metal ion was also employed for biomaterial modification. Han et al. used lithium chloride to enhance cementoblast differentiation potential of PDLSCs. Local injection of lithium chloride could activate Wnt signaling pathway and upregulate gene expression of cementum-related markers including osteocalcin (OCN), osteopontin (OPN), cementum protein 1 (CEMP1), and cementum attachment protein (CAP), further promote cementogenic differentiation of PDLSCs *in vitro* [28].

However, the use of a single biological factor has not proven sufficient to fully enhance the multi-directional differentiation potential of PDLSCs. As a result, the combination of multiple biological factors has been extensively researched. For instance, the combined use of calcium phosphate cement (CaP) and FGF-2 exhibited a 3.3-fold increase in PDL healing scores and a 1.9-fold increase compared to the use of CaP alone. Besides, the combination of CaP and BMP-2 could promote alveolar bone regeneration but could not enhance periodontal complex regeneration [29]. However, maintaining the long-term presence of growth factors in the local area often requires high doses, leading to increased costs and a higher risk of side effects. Moreover, periodontal tissue regeneration is a complex process regulated by multiple factors, making it challenging to determine the optimal type and quantity of biological factors needed throughout the regeneration process. The potential synergistic and antagonistic effects between these factors cannot be overlooked either (Table 1).

**Table 1:** The modification strategies enhancing multi-directional differentiation of PDLSCs.

Modification strategies		Application in periodontal regeneration	Limitations	Ref.
**Modulate the topography of biomaterials**				
mnHA		Enhance the attachment, spreading, proliferation, ALP activity, and cementogenic differentiation of PDLSCs	Limited regulatory effect on stem cells Intricate and complex fabrication of the materials Ethical and safety issues	[33]
Nanostructured F/CAP-coated MEW scaffold		Upregulate osteogenic genes in PDLSCs		[34]
PDLSCs bioprinted in GelMA of different concentrations		High-concentration GelMA hydrogel promote PDLSCs' osteogenic differentiation		[35]
Bioprinting PDLSC- laden collagen scaffold		Preserve cell viability, stimulate PDLSC differentiation and migration, form a well-defined parallel alignment		[36]
**Modify biomaterials with a single bioactive factor**				
Cytokine	BDNF	Stimulate the formation of new alveolar bone, cementum and connective new fibers inserted into the newly formed cementum and bone	Could not fully enhance the multi-directional differentiation potential of PDLSCs	[37]
	rhBMP-2	Promote the formation of new cementum with Sharpey's fibers on the instrumented root surface		[38]
	IL-33	Stimulate proliferation, clonogenicity and expression of pluripotency markers of PDLSCs		[39]
Metal ion	Lithium	Enhance cementoblast differentiation potential of PDLSCs Upregulate gene expression of cementum-related markers *in vitro* Activate Wnt signaling pathway		[28]
**Modify biomaterials with the combination of multiple biological factors**				
CaP and FGF-2		Promote the PDL healing in rats	Costs and risk of side effects because of the high does Could not overlook potential synergistic and antagonistic effects between multiple factors	[29]
CaP and BMP-2		Promote alveolar bone regeneration in rats		[29]
SDF-1/EX-4		Facilitate the proliferation, migration and osteogenic differentiation of PDLSCs *in vitro* Promote periodontal bone regeneration in rats		[141]
SDF-1α and PTH		Promote proliferation, migration and osteogenic differentiation of PDLSCs *in vitro*		[142]
bFGF and BMP-2		Promote the angiogenesis of PDLSCs Promote osteogenic differentiation of PDLSCs *in vitro* Facilitate satisfactory periodontal bone regeneration in rats		[48]

Regrettably, an ideal modification strategy which could simultaneously satisfy the multi-lineage differentiation of PDLSCs has still not been developed. A closer examination of the biological process of periodontal regeneration reveals that the implantation of biomaterials will inevitably activate immune response with multifarious activated cells and released molecular factors. Unidirectional regulatory strategy cannot fully stimulate the function of PDLSCs and a more comprehensive modulatory strategy should be introduced. The research strategy has shifted from single directional differentiation strategy to a more comprehensive immune microenvironment regulation.

### 3.2 Periodontal regeneration strategy based on immunomodulated manipulation of PDLSCs

As a foreign body, the implantation of biomaterials will inevitably activate immune response. The biological function of PDLSCs is regulated by the immune microenvironment. The activation of local and systemic immune response plays a crucial role in the construction of immune microenvironment. Our previous researches have shown that a favorable immune microenvironment has the potential to synergistically regulate periodontal multi-tissue regeneration events, including alveolar bone regeneration, periodontal ligament regeneration and cementum regeneration.

There is a strong relationship between immune cells and PDLSCs. Among the immune cells, macrophages play an important role in influencing PDLSCs’ behavior, primarily through the release of cytokines and growth factors. Following tissue injury, macrophages could attract PDLSCs to the site of damage *via* chemotactic signals, facilitating the recruitment of PDLSCs [40]. In the early inflammatory phase, M1 macrophages release pro-inflammatory cytokines like TNF-α, IL-1β and IL-6, creating a pro-inflammatory environment that hinders the regenerative potential of PDLSCs. Conversely, M2 macrophages release growth factors such as TGF-β and IL-10 to resolve inflammation and stimulate proliferation and differentiation of PDLSCs, contributing to tissue regeneration [41,42]. M2 macrophages could secrete VEGF and other pro-angiogenic factors that could support the formation of blood vessels [43].

Besides, T cells participate in the immune regulation of PDLSCs’ biological function. In periodontitis, T helper cells (Th1, Th17) and cytotoxic T lymphocytes (CTLs), secrete pro-inflammatory cytokines like TNF-α, IFN-γ, IL-17 and IL-22, inducing apoptosis in PDLSCs and contributing to tissue destruction [[44], [45], [46]]. The inflammatory cytokines released by T cells might alter the PDLSCs niche, impairing the capacity for differentiation [47]. Nevertheless, regulatory T cells (Tregs) have a protective role in tissue homeostasis and regeneration by creating a more favorable environment. Tregs could release anti-inflammatory cytokines such as IL-10 and TGF-β to modulate the inflammatory environment, prevent chronic inflammation and promote PDLSCs survival and function.

**Fig. 3:**
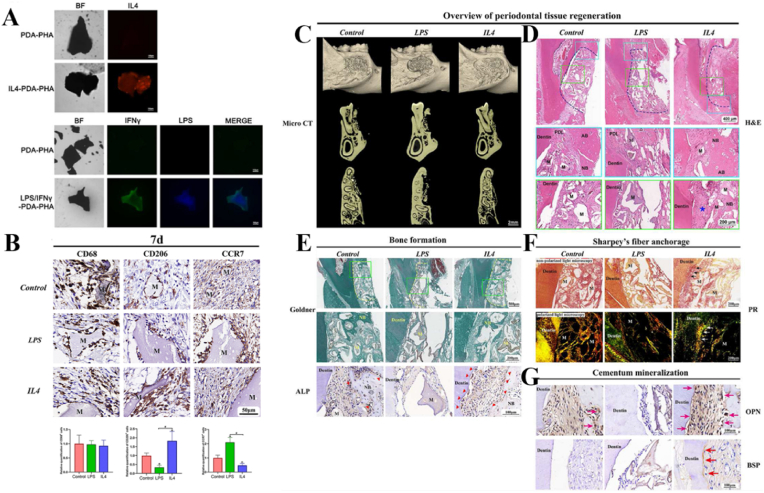
**Periodontal regeneration strategy based on immunomodulated manipulation of PDLSCs** [10]. (A) The immunofluorescence staining shows the loading of IL4 and LPS/IFNγ on the surface of PDA-PHA particles respectively. (B) IHC staining image and quantitative analysis of the CD68^+^, CD206^+^, and CCR7^+^ cells at day 7. (C–D) Three-dimensional reconstruction of the micro-CT and H&E staining results shows the overview of the periodontal regeneration outcome. Better alveolar bone regeneration was observed in the control and IL4 groups. (E) The bone formation effect was evaluated using Goldner's Masson trichrome (Goldner) and ALP IHC staining. Goldner's staining enables the visualization of the different degrees of mineralized tissue, staining newly formed bone and collagen fibers in dark and light green. (F) Sharpey's fiber formation was observed *via* PR staining using non-polarized/polarized light microscopy. (G) The mineralization of cementum and the anchorage of Sharpey's fiber on the dental root side was observed *via* OPN and BSP IHC staining. M: materials; NB: new bone; AB: alveolar bone; PDL: periodontal ligament; ∗: Sharpey's fiber.

Certain bioactive factors and trace elements crucially regulate macrophages to promote periodontal regeneration. For example, IL-4 and IFN-γ can be loaded onto hydroxyapatite for programmed release, inducing macrophage phenotypic transition to regulate inflammation and promote periodontal repair in our previous research [10] (Fig. 3). Ding et al. developed a fibrous scaffold sequentially releasing basic fibroblast growth factor (bFGF) and bone morphogenetic protein-2 (BMP-2), promoting PDLSCs angiogenesis and macrophage polarization to the pro-healing M2 phenotype, thus modulating inflammation [48]. Wu et al. demonstrated that fluorinated porcine hydroxyapatite (FPHA) elicited significant osteoimmunomodulatory effects in modulating inflammatory cytokines and osteogenic and angiogenic factors, promoting osteogenesis and angiogenesis [49]. Neutrophils also play a key role in periodontal microenvironment homeostasis and may represent a novel therapeutic target. Liu et al. found that Litcubanine A (LA) effectively inhibits neutrophil chemotaxis and suppresses the expression of genes associated with neutrophil respiratory burst and inflammation [50]. Additionally, the use of electroacupuncture (EA) suppressed neutrophil infiltration and relieved periodontal bone resorption by modulating pro- and anti-inflammatory cytokines in periodontal tissues [51].

Currently, most studies focus solely on the effect of biomaterials on the local microenvironment after implantation, often overlooking their effect on systemic immunity. Kaitlyn Sadtler et al. have demonstrated that the implantation of tissue-derived extracellular matrix scaffolds into a mouse muscle defect model led to an increase in local M2 macrophages, Th2 cells, and enhancement in local regeneration. At the same time, the increased number of Th2 cells in blood and the activation of immune activity in draining lymph nodes can also be observed [52]. Kelly M et al. improved the hydrophilicity of titanium implants through performing surface treatment, which resulted in the upregulation of Th2 cell and, anti-inflammatory factors such as IL4, IL10, IL13 in the experimental group, thereby enhancing the effect of tissue regeneration [53]. An increase in the number of Th2 cells in the spleen was also observed. Therefore, elucidating the systemic immunomodulatory properties of biomaterials is of great significance for deepening the understanding of the immunomodulatory properties of biomaterials and constructing biomaterials with enhanced immunomodulatory properties.

In summary, an ideal immune microenvironment could comprehensively regulate the biological function of PDLSCs, including proliferation, migration, osteoblast differentiation and cementoblast differentiation. Following implantation, biomaterials initially interact with blood and activate the coagulation cascade, leading to the formation of a biomaterials-clot complex. This complex serves as the primary barrier between the biomaterials and immune cells, thereby influencing the subsequent immune microenvironment. Therefore, with the advancement of research on immunomodulatory biomaterials, the intermediary role of blood in biomaterial-mediated tissue regeneration process has further attracted much attention.

### 3.3 Periodontal regeneration strategy based on coagulo-immunomodulatory manipulation of PDLSCs

Blood coagulation and initiation of inflammation are the most typical early events in periodontal tissue regeneration, with considerable overlap between the early immune microenvironment and the blood coagulation process. Blood clots naturally contain a variety of cells, such as neutrophils, macrophages, T cells and other immune cells that participate in the regulation of tissue regeneration [11,12], while mesenchymal stem cells play a role in immune regulation [54]. Biomaterials could absorb fibrinogen, coagulation factor and other components from the remaining blood, resulting in changes in the fibrin network and surface fibrin films within the clot [55]. The change of physicochemical properties of blood clot fibrin will influence the immune microenvironment, thereby affecting the efficiency of tissue regeneration [56,57]. The blood clots interact with immune cells in multiple ways, playing a crucial role in shaping the immune microenvironment [13,14].

In recent years, the interaction between biomaterials and blood has attracted extensive attention. It has been found that when biomaterials contact with blood, they alter the structure of blood clot, which plays a crucial role in regulating the immune microenvironment of the local defect. The changes of fibrin network induced by biomaterials have a double-sided effect on tissue regeneration: they can exacerbate the foreign body reaction and impair tissue regeneration, or they can mitigate the foreign body and inflammatory responses by rearranging the fibrin network, thereby enhancing tissue regeneration [56]. The blood clots can act on immune cells in several ways, contributing to the shaping of the immune microenvironment [13,14].

At present, various studies have been conducted to modify the structure of blood clots to regulate the immune microenvironment. In this section, the regulation mechanism of biomaterials on coagulation-immune complex will be introduced from three aspects: fiber protein modification, fiber mesh binding with exogenous protein modification and fibrin clot morphology.

Modifying fibrin can regulate the biological properties of the fibrin network. Some studies investigated the role of matrix stiffness in the regulation of macrophage activity by manipulating the mechanical properties of fibrin utilizing a photo-initiated crosslinking method [58]. These studies have observed that matrix crosslinking induces distinct changes in macrophage morphology, integrin expression, migration, and inflammatory activation. Additionally, modification of fibrinogen with lysine-reactive polyethylene glycol can effectively increase the crosslinking degree of fiber network [15].

Integrating functional proteins with the fibrin network to enhance its biological characteristics is another important regulatory strategy. Hyaluronic acid (HA) is a modified material that regulates the morphology of fiber network. High molecular weight HA (1500 kDa and 500 kDa) can increase fiber diameter and enhancing fibrin network porosity, while low molecular weight HA (25 kDa) can reduce fiber diameter [59]. Fibrin/HA coating can significantly enhance initial cell adhesion at the scaffold interface [60].

The microporous structure of biomaterials can trap blood fibrinogen and coagulation factors, altering the structure of blood clot fibrin network and films to regulate the immune microenvironment. The blood fibrinogen is captured by the size effect of mesoporous materials, and then the *in-situ* assembly and morphology of the fibrin network are regulated. Mesopore with size greater than 9 nm can enrich fibrinogen, increases the nucleation site of coagulation process, and mediates the generation of finer diameter fibrin network [13]. This delicate network of fibrin regulates cell adhesion, cytoskeletal assembly and induces a minor inflammatory response in macrophages, ultimately influencing the level of inflammation following cell adhesion. The particle size of porcine bone-derived hydroxyapatite (PHA) can manipulate the microporous structure, thus regulating the absorption of blood fibrinogen and coagulation factors and the subsequent formation of fibrin films [14]. PHA with more microporous structures adsorbed more coagulation factors, resulting in the formation of thinnest and sparsest fibrin films. These thinnest and sparsest fibrin films increased inflammation and profibrosis of macrophages, leading to the stronger foreign body reaction (FBR) (Table 2).

**Table 2:** Coagulo-immunomodulatory strategies on biomaterials.

Regulation manners	Affects on coagulation and immune modulation	Ref.
**Fiber protein modification**		
Manipulate mechanical properties of fibrin	Induce distinct changes in macrophage morphology, integrin expression, migration, and inflammatory activation	[58]
Modify fibrinogen with lysine-reactive polyethylene glycol	Increase the crosslinking degree of fiber network	[15]
**Fiber mesh binding with exogenous protein modification**		
Binding with high molecular weight HA	Increase fiber diameter and enhance fibrin network porosity	[59]
Binding with low molecular weight HA	Reduce fiber diameter	[59]
Fibrin/HA coating	Enhance initial cell adhesion at the scaffold interface	[60]
**Fibrin clot morphology modification**		
Modify microporous structure of biomaterials	Enrich fibrinogen, increase the nucleation site of coagulation process and mediate the generation of finer diameter fibrin network	[13]
Modify particle size of PHA	Regulate the absorption of blood fibrinogen, coagulation factors and the subsequent formation of fibrin films	[14]

Although the coagulo-immunomodulatory strategy has demonstrated some success, our research indicates that this approach only leads to partial regeneration of periodontal tissue, with overall periodontal regeneration outcomes remaining suboptimal. Achieving ideal periodontal complex regeneration has proven challenging. The biological essence of these strategies lies in promoting the multidirectional differentiation of PDLSCs. However, clinical trials involving PDLSCs *in vivo* have not met the anticipated improvements in periodontal regeneration outcomes, prompting a critical reevaluation.

**Fig. 4:**
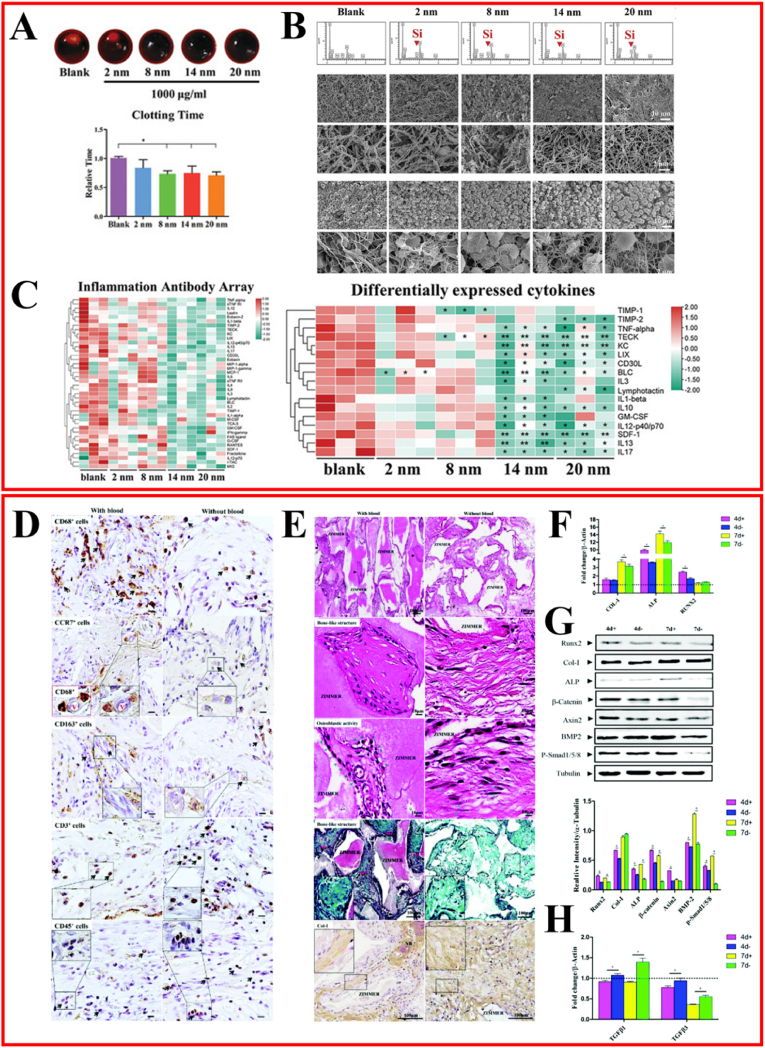
**Tissue regeneration strategy based on coagulo-immunomodulation** [13,56]. (A) Coagulation time test of different mesopore size *in vitro*. (B) Element distribution of mesoporous silica‐regulated fibrin network using SEM‐EDS. SEM observation of mesoporous silica‐regulated fibrin gel and blood clot fibrin network. (C) Cytokine profiles of cell cultured supernatant of macrophages cultured on fibrin network using Inflammation Antibody Array. (D) Representative CD68^+^, CCR7^+^, CD163^+^ macrophage and CD3, CD45 IHC staining of tissue sections from subcutaneous implants at 6 weeks post-surgery. (E) H&E staining, Safranin O staining and IHC staining for Type I collagen (Col-I) of tissue sections at 6 weeks post-subcutaneous implantation. (F) RT-qPCR analysis of osteogenesis-related genes in samples collected 4 d and 7 d post-subcutaneous implantation: ALP, Col-I, and Runx2, which were mostly upregulated. (G) Western blot results of osteogenesis-related proteins (Runx2, Col-I, ALP) and osteogenesis-related signaling pathways (β-catenin, Axin2, BMP-2, and p-Smad1/5/8). (H) RT-qPCR analysis of fibrosis-related gene (TGF-beta 1/3) expression levels, which were mostly upregulated in the blood (−) group. M: HA/TCP; NB: newly formed bone. ∗p < 0.05.

As periodontal tissue develops and matures, the functional activity of PDLSCs significantly diminishes, evidenced by a decreased number and weakened proliferation, migration, and differentiation capabilities. Therefore, stimulating the function of PDLSCs to achieve periodontal complex regeneration remains challenging. The biological foundation of periodontal regeneration requires updating and innovation. By examining the developmental process of the periodontal complex, we are motivated to advance the next generation of periodontal regeneration strategies, inspired by the concept of periodontal developmental engineering (Fig. 4).

## 4 The next generation of periodontal regeneration strategy: periodontal developmental engineering

Developmental engineering is an emerging approach within regenerative medicine that draws inspiration from the principles of developmental biology to guide the regeneration of tissues and organs [61,62]. It aims to mimic the natural processes that occur during embryonic development in order to create functional tissues and organs. Unlike traditional regenerative medicine, which often relies on replacing damaged tissues with engineered substitutes or using stem cells to repair tissues, developmental engineering seeks to replicate the developmental processes themselves. This approach allows for a more natural and potentially more effective regeneration of complex tissues. Thus, developmental engineering aims to regenerate the entire periodontal complex, including the periodontal ligament, alveolar bone, and cementum, rather than just repairing individual parts. This holistic approach is necessary for restoring both the function and structure of the periodontium [63]. Approaches such as recreating the odontogenic microenvironment and precisely delivering growth factors and signaling molecules at specific stages of the regenerative process to mimic the natural developmental cues hold great promise. However, significant differences exist between developmental individuals and adults. Thus, it is necessary to review the developmental process of periodontal complex and dissect the differences between developmental and regenerative processes.

### 4.1 The difference between developmental and regenerative processes

The development of periodontal tissue begins with the induction of Hertwig's epithelial root sheath (HERS) and ends with the closure of the apical foramen. Key developmental events such as epithelial induction, PDL fiber anchoring, growth, and mineralization are all involved in this complex process. Dental follicle cells (DFs) derived from the mesenchymal layer of the ectoderm, which exhibit typical fibroblast-like cell morphology, differentiate into osteoblasts, fibroblasts, and osteoblasts when the apical root sheath ruptures during the bell shaped phase. Their positional relationships guide this differentiation, leading to the formation of cementum, periodontal ligament, and alveolar bone, thereby establishing the periodontal complex [27,64]. Upon tissue maturation, DFs ultimately evolve into PDLSCs and HERS degenerates into the remnants of the Malassez epithelial rest. The dysfunction of DFs and the disappearance of HERS signal increase the difficulty of periodontal complex regeneration in adults.

#### 4.1.1 Stem cells involved in periodontal development and regeneration

In the developmental process, the dental follicle eventually evolves into a population of periodontal ligament cells (PDLCs). During periodontal development, PTHrP^+^ dental follicle cells have been uniquely identified as key contributors to the periodontium, serving as a major cellular source for diverse cell types including PDL cells, cementoblasts, and alveolar bone osteoblasts [65]. However, the PTHrP^+^ DF cells disappear in mature periodontium. Although a small amount of PDLSCs remains in mature periodontium, their functional activity declines significantly, which is manifested as decreased number, as well as weakened proliferation, migration and differentiation activity. Researches on canine periodontium has indicated that aging influences the functional changes of PDLCs and cementoblasts, increasing markers of senescence, chronic inflammation, and apoptosis [66]. Similarly, human PDLSCs obtained from elderly donors demonstrated a diminished capacity for both proliferation and differentiation compared with those from younger donors. Furthermore, after cultured with conditioned medium from aged PDLSCs, there was a notable reduction in young PDLSCs’ ability to form connective tissues, as well as osteogenesis and cementogenesis capabilities [67].

**Fig. 5:**
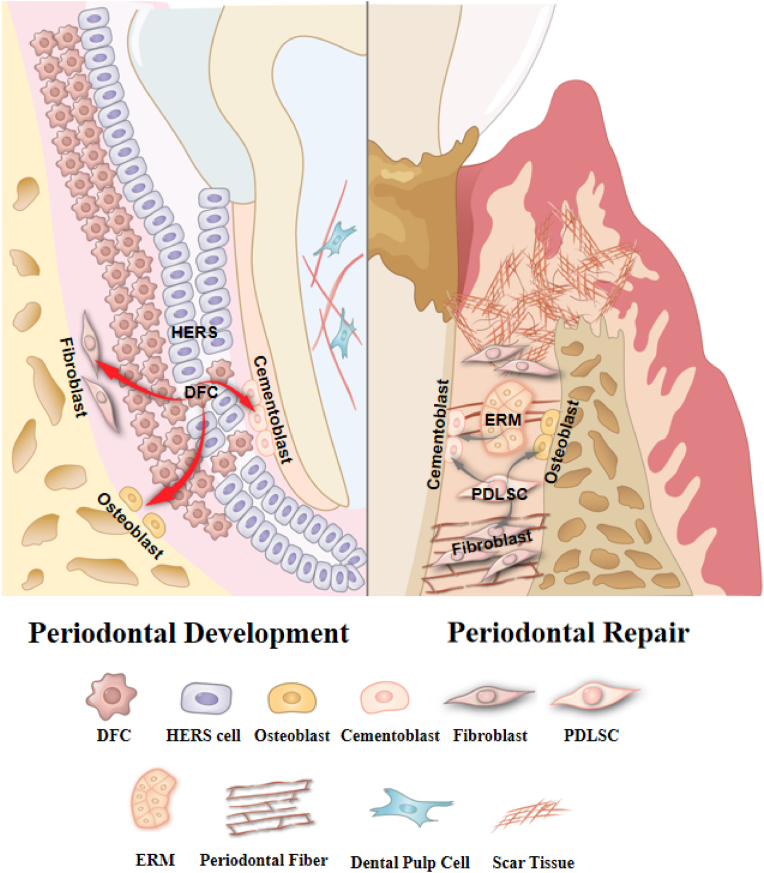
**Different cells involved in periodontal development and repair process.** There are significant differences in cell types between periodontal developmental process and repair process. During development, DFCs and HERS are primary sources of fibroblasts, cementoblasts and osteoblasts, with great potential for differentiation. In contrast, during repair process, a small number of PDLSCs and ERM exhibits limited stemness, accompanied by decreased proliferation, migration and differentiation capacities.

Besides, accumulating evidence suggests that in the periodontal development, HERS cells are recognized as a significant source for cementoblasts and key developmental signals for root elongation. An *in vitro* study has demonstrated that HERS cells initially engage in the synthesis and secretion of specific enamel-associated proteins. Subsequently, these cells undergo morphological alterations and produce a mineralized extracellular matrix resembling acellular cementum, indicating their potential to differentiate into cementoblasts. The combination of dental follicle cells and the HERS cells gave rise to increased cementum-like and periodontal ligament-like structures after transplantation into rats' omenta, while the control group only produce fibrous tissues [68,69]. Moreover, HERS cells generate distinctive signals that regulates the orchestration of mesenchymal cells, which would be further illustrated below. In mature periodontium, HERS undergoes apoptosis, with the remnants residing within the developed periodontal ligament as the epithelial cell rests of Malassez (ERM). Studies have reported that ERM exhibits stem cell-like properties, capable of generating bone, cementum-like, and Sharpey's fiber-like structures after transplantation into immunocompromised mice [70]. In porcine, after co-culturing with dental pulp cells, ERM can differentiate into ameloblast-like cells and generate enamel-like tissues *in vivo* [71]. These inspiring results indicates that ERM is of great significance in PDLSCs-based periodontal regeneration. However, the regenerative capacity of ERM is relatively limited. In cases of substantial defect on the tooth root surface, the body tends to directly fill the gap through osteogenesis, ultimately leading to dental ankylosis without restoration of functional cementum or periodontal ligament structures [72] (Fig. 5).

#### 4.1.2 Distinct microenvironments between periodontal development and regeneration

There are significant microenvironment differences between developmental and regenerative processes in periodontal tissue. This is primarily reflected in the differences in the expression of epithelial-derived molecules and the distinct immune microenvironments. During periodontal development, epithelial-derived signaling molecules from HERS such as β-catenin, Wnt, FGF and Shh interact with the dental follicle to orchestrate the tooth root extension and periodontal complex formation. For example, the inactivation of β-catenin leads to early premature disruption of HERS, resulting in decreased cell adhesion as well as expression of junctional proteins. Conversely, the stabilization of β-catenin prevents the early interruption of HERS, maintaining the cohesion of HERS cells and increasing the expression of junctional proteins [73]. Additionally, Nemoto et al. have illustrated that HERS possesses the capability to induce the differentiation of dental follicle cells into cementoblasts or osteoblasts through Wnt/β-catenin signaling pathway. Moreover, previous research highlighted the critical role of the Shh signaling pathway in regulating epithelial-mesenchymal interaction [74]. Huang et al. has discovered that HERS cells regulate the dental mesenchyme through the Smad4-Shh-Nfic pathway. [75]. Regarding FGF signaling, FGF-10 plays a crucial role in the balance of crown and root development. Overexpression of FGF-10 inhibits HERS formation, while the loss of FGF10 signaling can shift the focus from tooth eruption to root extension [76]. However, some of these epithelial-derived signals are absent during regenerative process.

On the other hand, the immune microenvironments surrounding periodontal tissues during the developmental and regenerative phases are different. In the regenerative processes, the immune response is more intensely activated compared with the developmental process. Besides, innate immune response plays a crucial part in maintaining tooth development while the adaptive immune response remains highly active during the immune periodontal regeneration process. During development, the immune response is relatively subdued compared to the regenerative phase. The immune cells are more focused on maintaining tissue homeostasis and supporting the developmental processes rather than actively combating inflammation [77]. Developmental processes are regulated by specific signaling pathways that are crucial for tissue formation and remodeling. For instance, the MyD88/p38 MAPK pathway plays a significant role in periodontal remodeling during development, influencing tissue development and immune response modulation [78]. A study comparing the transcriptome landscape of periodontium in developmental and renewal stages found that the developing periodontium (DeP) showed a downregulation of immune response, with activation, migration, and recruitment of immune cells being less pronounced compared to the regenerative phase. In the regenerative phase, the immune response is significantly more active. This is due to the presence of inflammation and the need to combat pathogens and promote tissue repair. The immune microenvironment is characterized by increased activity of leukocytes and inflammatory molecules. The regenerative environment involves a dynamic shift in immune cell populations. For example, macrophages play a crucial role in modulating the immune response. M1 macrophages promote inflammation and tissue breakdown, while M2 macrophages are involved in tissue repair and regeneration [79]. Therefore, even if the same signaling molecules expressed in developmental stage could be introduced, they may exert different functions due to the inflammatory condition in periodontal regeneration. Regulators such as β-catenin, BMP, IGF and FGF signaling pathways tend to promote tissue programming and patterning during development, but in regeneration, they primarily serve to enhance cell proliferation and tissue migration. Research showed that under inflammatory condition, β-catenin activated by Wnt3a could promote proliferation of PDLSCs but suppress the capability of osteogenic differentiation [80]. BMP was previously considered to play a pivotal role in the regulation of tooth eruption and root formation, with higher expression levels in dental follicle than in the coronal region [81]. Results from scRNA-seq showed that the BMP4/2-BMPR2 pathway has regulatory potential over osteoclast genes like SPP1 and GREM1 [82]. As for periodontal repair, results showed that BMP2 enhances bone generation and cementogenesis in periodontal defect, but with unhinged periodontal relationship [83,84]. Similarly, the paracrine expression of IGF‐1 mediates root elongation, for the growth of the root sheath was observed in mouse HERS organ treated with IGF-1. While, in periodontal regeneration, IGF1 is implicated in stimulation of the proliferation and differentiation of osteoblasts [85,86]. Continuous layer of osteoblasts lined the newly formed bone was observed with IGF-1 treatment *in vivo*, while functional Sharpey's fibers were absent [87].

Besides, during periodontal development, the sequential regulation of various bioactive signals maintains a dynamic homeostasis, while the equilibrium is disrupted in periodontal repair due to the change of immune microenvirnment. To be specific, the OPG/RANKL axis helps balance the osteogenesis and osteoclasis in periodontium. However, the balance is destroyed in an inflammatory environment within periodontal defects. Under physiological conditions, mature periodontal ligament tissue is rich in OPG that plays a role in suppressing the formation of osteoclasts. However, during the process of tooth eruption, there is a temporary reduction in OPG production, which facilitates osteoclastogenesis, a crucial developmental event for root formation [88]. Once tooth eruption is completed, the ratio of OPG/RANKL is restored to maintain periodontal stability. Moreover, amelogenin and its product LRAP have been suggested to modulate the OPG/RANKL axis [89]. Consequently, they serve as a safeguard against the resorption of the root surface mediated by osteoclasts. In the context of the periodontitis microenvironment, there is persistent upregulation of RANKL and decrease in OPG levels, thereby promoting the occurrence of osteoclast formation and resulting in continuous osteoclasis [90] (Table 3).

**Table 3:** Different microenvironments between periodontal development and regeneration.

	Developmental processes	Regenerative processes
**Epithelial-derived signaling molecules**		
Wnt/β-catenin signaling	Regulate interruption of HERS Induce the differentiation of dental follicle cells into cementoblasts or osteoblasts	Lack of some epithelial-derived signals
Shh signaling	Regulate epithelial-mesenchymal interaction	
FGF signaling	Balance crown and root development	
**Immune microenvironments**		
Immune response	Relatively less-activated	Relatively over-activated
	Innate immune response plays a crucial part	Adaptive immune response remains highly active
	Less pronounced activation, migration, and recruitment of immune cells	Increased activity of leukocytes and inflammatory molecules
	Immune cells more focus on maintaining tissue homeostasis and supporting the developmental processes	Immune cells more focus on combating inflammation
Functions of signaling molecules	Promote tissue programming and patterning	Enhance cell proliferation and tissue migration
	BMP plays a pivotal role in the regulation of tooth eruption and root formation, with higher expression levels in dental follicle	BMP2 enhances bone generation and cementogenesis in periodontal defect, but with unhinged periodontal relationship
	IGF‐1 mediates root elongation	IGF-1 stimulate proliferation and differentiation of osteoblastsfunctional with Sharpey's fibers absent
Equilibrium of bioactive signals	Temporary reduction in OPG production to facilitate osteoclastogenesis during the process of tooth eruption	Persistent upregulation of RANKL and decrease in OPG levels, promoting the occurrence of osteoclast formation

### 4.2 The research progress and application of periodontal developmental engineering

Developmental engineering draws inspiration from the morphogenetic processes that occur during embryonic development. By replicating these processes, it aims to generate tissues and organs with correct biomorphology and biofunctionality. It integrates principles from developmental biology, materials science, and engineering to design strategies that closely resemble the natural environment and cues required for tissue regeneration. Compared to other methods, developmental engineering strategy offers unique advantages. The development of periodontal tissue engineering primarily involves two aspects. The first involves the application of cells, which includes the direct use of stem cells that participate in periodontal formation during development, inducing cells to revert to a developmental state, and promoting mesenchymal aggregation. The second aspect focuses on simulating the periodontal developmental microenvironment, which encompasses the induction of epithelial-derived signals, the application of extracellular matrix and the modulation of the immune microenvironment.

#### 4.2.1 Perform developmental engineering on the cellular aspect

Direct application of cells harvested from developmental process has emerged as a strategy to achieve a developmental state in regenerative efforts. These specific cells, which play crucial role in development period, hold great potential for periodontal regeneration. (1) Embryonic stem cells (ESCs), derived from the undifferentiated inner cell mass of the blastocyst, have the ability to differentiate into various cell lineages, including endoderm, mesoderm and ectoderm [91]. ESCs possess the potential to differentiate into fibroblasts and osteoblasts, facilitating the generation of tooth-periodontium complex structures [[92], [93], [94], [95]]. ESCs could also improve periodontal regeneration in furcation defects in porcine [96]. (2) Dental follicle cells (DFCs) have the capacity to differentiate into periodontal ligament cells, osteoblasts and cementoblasts, thereby contributing to the formation of periodontium complex. DFCs, being the closest to periodontal developmental state, holding great potential to regenerate cementum-PDL tissues [97]. DFCs cell sheets could induce cementum- and PDL-like tissue formation through epithelial-mesenchymal interactions (EMT) [68,98]. When combined with treated dentin matrix (TDM), DFCs stimulate periodontal complex regeneration in artificial dental roots [97,99]. The underlying mechanism of periodontal regeneration of DFCs application might be related to periostin-mediated macrophages reprogramming [100]. The whole dental follicle tissues collected from mice at ED18.5 could generate a functional periodontal complex when loaded onto the surface of titanium with hydroxyapatite coating [101]. (3) Stem Cells from Human Exfoliated Deciduous Teeth (SHEDs) demonstrated their developmental potential, with different arrangements of fiber in the cervical, middle and apical part of the root and fibers anchoraged in the cementum. Additionally, SHEDs have shown promise in treating with periodontitis by reducing bone loss and suppressing inflammation, due to the ability to induce M2 macrophage phenotype [102,103]. When transplanted *in vivo*, SHEDs might promote periodontal bone regeneration through regulating the microenvironment and recruiting osteoblasts [104]. (4) Stem Cells from the Dental Apical Papilla (SCAPs) enhanced periodontal tissue regeneration in a minipig model of periodontitis *via* local injection [105]. Another study demonstrated that SCAPs, combined with PDLSCs cell sheets could achieve functional tooth regeneration [106]. However, there are certain limitations to the direct use of developmental cells, including immunological rejection and lack of interaction with the mature organism. Moreover, the optimal choose of the state of development cells remains unclear. ESCs possess stronger stem cell properties, while DFCs, SHEDs, and SCAPs exhibit more periodontal-specific properties.

Another promising approach to periodontal regeneration is inducing cells to revert to developmental state. Induced pluripotent stem cells (iPSCs) have garnered substantial interest in regenerative medicine for years. iPSCs could be reprogrammed to differentiate into various cell types, including endoderm, mesoderm and ectoderm, thus achieving a form of “re-development” [107]. Unlike ESCs, iPSCs bypass significant ethical concerns and exhibit greater ability of proliferation and differentiation. In periodontal regeneration, iPSCs have been successfully reprogrammed from various kinds of dental tissues. For instance, iPSCs reprogrammed from gingival fibroblasts possessed the ability of periodontal differentiation when induced by EMD or GDF-5 [108]. Additionally, iPSCs could differentiate into periodontal ligament stem cells (PDLSCs)-like cells, when exposed to extracellular matrix secreted by PDLCs [109]. *In vivo* studies have shown that iPSCs combined with EMD or BMP6 hydrogel exhibited great potential to improve formation of alveolar bone, PDL and cementum to promote regeneration in periodontal defect [110,111]. In addition, iPSCs could be reprogrammed into iPSC-derived neural crest-like cells (NCLCs) and iPSC-derived mesenchymal stem cells (iPSC-MSCs) [112,113]. It was reported that iPSC-MSCs have shown considerable efficacy in periodontal regeneration both *in vitro* and *in vivo* [111,[114], [115], [116]].

Thirdly, the application of mesenchymal aggregation has also been proposed. During developmental process, mesenchymal progenitors or mesenchymal stem cells (MSCs) possess an intrinsic property to form compact spheroid-like assemblies, a phenomenon called “mesenchymal aggregation”. Mesenchymal aggregation has been known as a necessary component initiating organ buds and a center of organ development [117]. A thorough understanding of mesenchymal aggregation in the developmental process, along with innovative construction and application of mesenchymal aggregation has been a promising trend for the regenerative medicine. Significant strides have been made in exploring the application of mesenchymal aggregation in periodontal regeneration. For instance, human periodontal ligament stem cells (hPDLSCs) and human umbilical cord mesenchymal stem cells (hUCMSCs) were respectively used to constructed cell aggregates, showing contribution to regeneration of both soft and hard periodontal tissues under periodontitis condition [118]. Another study on PDLSCs aggregates demonstrated that resveratrol could enhance the functionality and regeneration capabilities of mesenchymal stem cell aggregates [119]. In addition, it was reported that tissue-specific composite aggregates of PLDSCs and bone marrow-derived mesenchymal stem cells (BMMSCs) drived periodontium tissue regeneration by reconstructing a regenerative microenvironment [120]. Mesenchymal aggregation offers the advantages of better biocompatibility and realizes the interaction with other lineage progenitors (Table 4). Though the mesenchymal aggregation has hit a milestone, it should be noted that the physiological, biochemical and biophysical mechanism underlying mesenchymal aggregation remain unclear [117]. With the advancements of the developmental engineering in the regenerative medicine in the future, the application of mesenchymal aggregation is worth looking forward to.

**Table 4:** Developmental engineering on cellular aspect.

Cell Types	Source	Differentiation Potential	Application in Periodontal Regeneration	Ref.
**Directly apply cells harvested from developmental process**				
ESCs	Inner cell mass of blastocyst stage embryos	Differentiate into any cell types of the adult organism	Facilitate the generation of tooth-periodontium complex structures	[[92], [93], [94], [95]]
			Improve periodontal regeneration in porcine	[96]
DFCs	Dental follicle	Differentiate into osteoblasts, fibroblasts, cementoblasts, neural cells and adipocytes	Regenerate cementum-PDL tissues in nude mice	[97]
			Induce cementum- and PDL-like tissue formation through EMT in rats	[98]
			Stimulate periodontal regeneration in artificial dental roots combined with TDM in rats	[99]
			Generate a functional periodontal complex in murine	[101]
SHEDs	Dental pulp of exfoliated deciduous teeth	Differentiate into neural cells, adipocytes, osteoblasts and odontoblasts	Form different arrangements of fiber in the different parts of the root and fibers anchoraged in the cementum in a tooth replantation model	[102]
			Show promise in treating with periodontitis in rats	[103]
			Promote periodontal hard and soft tissue regeneration in miniature pigs and rats	[102,104]
SCAPs	Root apical papilla from developmental period	Differentiate into odontoblast-like cells, adipocytes, osteoblasts, chondrocytes and neural cells	Enhance periodontal tissue regeneration in a minipig model of periodontitis	[105]
			Achieve functional tooth regeneration combining with PDLSCs in swine	[106]
**Inducing cells to revert to developmental state**				
iPSCs	Derived from any differentiated cell types	Have a developmental potency comparable to ESCs	Possess the ability of periodontal differetiation when induced by EMD or GDF-5 *in vitro*	[108]
			Improve formation of periodontal tissues when combined with EMD or BMP6 hydrogel in rats	[110,111]
			iPSC-MSCs show efficacy in periodontal regeneration both *in vitro* and *in vivo*	[[114], [115], [116]]
**Mesenchymal aggregation: an intrinsic property of mesenchymal progenitors or MSCs**				
Mesenchymal aggregation	Compact spheroid-like assemblies during developmental process	Necessary component initiating organ buds and a center of organ development	Cell aggregates of hPDLSCs and hUCMSCs contribute to regeneration of periodontal tissues under periodontitis condition in rats	[118]
			Tissue-specific composite aggregates of PLDSCs and BMMSCs drive periodontium tissue regeneration in nude mice and minipig	[120]

#### 4.2.2 Perform developmental engineering strategy targeting microenvironment aspect

Epithelial-derived signals play a crucial role in guiding periodontal development. In fact, the clinical efficacy of enamel matrix derivative (EMD) confirmed the effectiveness of “developmental engineering” to some extent. EMD, extracted from the enamel tissues of developing porcine teeth, is mainly composed of enamel matrix proteins (EMPS), including amelogenin, enamelin and tuftelin. EMD aligns with the developmental engineering concept by providing developmental signals. Numerous clinical trials have shown that combining EMD with bone grafts has favorable periodontal restoration effect, and using EMD alone also achieves positive results [121]. For instance, Meritxell et al. randomly treated 52 patients with bone defects using EMD combined with bone grafts (EMD/BC) or EMD alone. After 1 year, the average probing depth (PD) in the EMD/BC group decreased by 3.14 ± 1.95 mm (39.6 %), while in the EMD group, the average PD decreased by 3.30 ± 1.89 mm (48.7 %). The study concluded that EMD treatment, regardless of the presence of bone grafts, led to statistically significant improvements at 12 months compared to baseline. In contrast, the combined approach did not show statistically significant additional benefits [122]. EMP, secreted by ameloblasts during enamel organ development, include amelogenins (Am), which constitute over 90 % of the total protein [123]. Studies in mice have identified related genes that could provide insights into EMP production and its regulatory role in periodontal tissue [124]. EMPs possess anti-inflammatory properties, promote PDLSC differentiation, accelerate osteogenesis and mineralization, and facilitate cementum formation, though the underlying mechanisms are not fully understood. Layla's research using the TGFβ receptor type-I kinase inhibitor SB431542 to block related signaling pathways suggested that TGFβ mediates part of the anti-inflammatory effects of EMD *in vitro* [125]. EMD can also stimulate highly expression of CEMP1 and CAP proteins, promoting osteogenic differentiation of hPDLCs, though with lower mineralization levels [126]. EMD can induce PDLSCs to differentiate into cementoblast-like cells, with several microRNAs, including miR-30a, exhibiting differential expression [127]. In addition, these effects may also be related to the inhibitory effect of EMP on the proliferation and migration of gingival epithelial cells [128].

Besides, extracellular matrix (ECM) plays an important role in both periodontal development and repair process. Utilizing ECM from developmental periods might be a unique manner to mimic the developmental environment. Application of decellularized extracellular matrix (dECM) of tissues or cells is an effective method to preserve biochemical, biological and biophysical signals present in the ECM environment and deliver signals to the regenerative area [[129], [130], [131]]. Guo et al. employed a combination of decellularized tooth matrix (DTM) with human dental pulp stem cell (hDPSC) aggregates to simulate the developmental microenvironment related to teeth, achieving effective functional regeneration in both pigs and patients with traumatic tooth loss [132]. Using dECM from developmental dental follicle as a bioink for 3D-bioprinting greatly enhanced the anchoring structures of the bone-ligament interface, well-aligned periodontal fibers, and highly mineralized alveolar bone [133]. As the understanding of underlying mechanism and application of the ECM at developmental periods advances, and as engineering of dECM improves, dECM is expected to play an important role in mimicking the developmental environment [134].

**Fig. 6:**
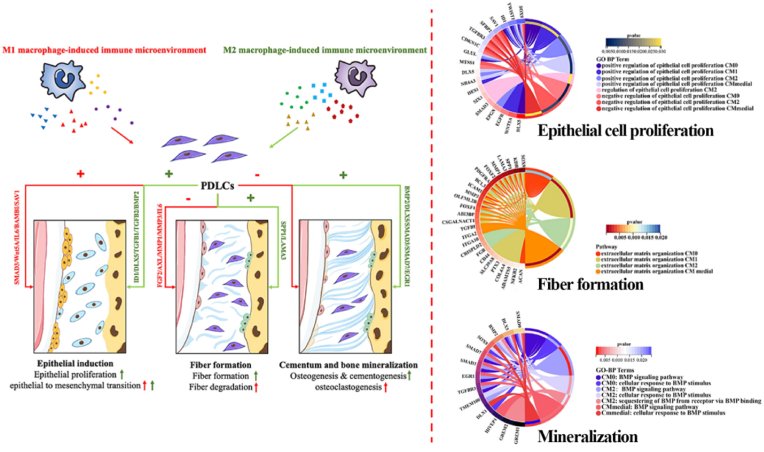
**The application of developmental engineering** [135]. Macrophage-induced immune microenvironment can arouse the periodontal mutlti-tissue developing potential of PDLCs. M1 type macrophage immune microenvironment promoted activities such as epithelial to mesenchymal transition, fiber degradation and osteoclastogenesis by certain molecular mechanisms. M2 type macrophage immune microenvironment enhanced activities such as epithelial proliferation, epithelial to mesenchymal transition, fiber formation and mineralization.

Thirdly, the immune microenvironment is also a target for the regulation and implementation of developmental engineering. Fine-tuning the microenvironments mediated by macrophages may hold promise for inducing the re-development state of PDLCs. Results from our research group demonstrated that immune microenvironment induced by M1-type macrophages promotes PDLCs activities such as epithelial-mesenchymal transition, fiber degradation, osteoclastogenesis, and inflammation. In contrast, the immune microenvironment induced by M2-type macrophages was superior in epithelial induction, fiber formation, and mineralization of PDLCs [135]. These findings indicated the potential of immune micro-environment regulation in performing the developmental engineering strategy (Fig. 6).

### 4.3 The clinical feasibility, challenges, and future of periodontal developmental engineering strategy

From the theoretical knowledge of developmental biology, tissue regeneration guided by developmental engineering strategies is currently the closest approach to achieve physiological regeneration. Developmental engineering has already achieved significant success in tissue regeneration in other fields. For example, in bone tissue engineering, the combination of BMSC-loaded hydrogel microspheres (MSs) with digital light-processing (DLP) printing technology, along with stepwise induction, has led to the development of osteo-callus organoids for rapid bone regeneration [16]. Our collaborative research with Jin etc combined decellularized tooth matrix (DTM) with human dental pulp stem cell (hDPSC) aggregates to simulate a developmental microenvironment related to odontogenesis. This approach successfully regenerated functional teeth in a preclinical pig model and was later tested in a pilot clinical trial involving 15 patients with avulsed teeth, showing promising results in regenerating functional teeth [132]. Meanwhile, a systematic review and meta-analysis of randomized controlled trials showed that the use of stem cells in tissue engineering for the treatment of periodontal defects has significant advantages over conventional therapies, particularly in the repair of clinical attachment loss (CAL) [136]. These studies suggest the feasibility of periodontal regeneration guided by developmental engineering.

However, the current clinical understanding of periodontal developmental engineering and clinical application remain limited, and numerous challenges persist when it comes to actual clinical translation. The first challenge involves the selection of developmental engineering seed cells and the construction of their microenvironment. A complete understanding of the specific signals and mechanisms involved in periodontal development is still lacking, which hinders the design of effective strategies that can accurately replicate the developmental microenvironment and the optimal choice of seed cells. The application of multi-omics methodologies, including single-cell technology, spatial transcriptome sequencing, and spatial proteomics, offers valuable insights into identifying vital cell types involved in developmental and regenerative processes. Multi-omics approaches not only identify target cell clusters that act as “seeds”, but also enable the dissection of the “soil”, which refers to immune microenvironment during development and regeneration. For example, through scRNA-seq, Gerber et al. discovered that the roles of epicardial cells and macrophages in the mouse heart injury response are highly stage-specific. Notably, the secretion of CLCF1 by neonatal-specific macrophages serves as a potent activator of cardiomyocytes proliferation [137]. Therefore, multi-omics technique enables the exploration of the transcriptomic and proteomic landscapes of both cells and microenvironments, providing new insights into developmental engineering.

Secondly, the use of stem cells, particularly embryonic stem cells, raises ethical concerns regarding their source and manipulation. Additionally, the potential risks, such as tumor formation, must be carefully considered. However, odontogenic stem cells derived from SHEDs in exfoliated deciduous teeth and DFCs from third molar tooth germs can effectively circumvent ethical concerns. These cells may represent a crucial source for future applications in periodontal tissue engineering. On the other hand, the use of artificial synthetic materials to induce somatic cells into iPSCs and to form mesenchymal condensates from MSCs represents a promising strategy in tissue engineering [138,139]. This approach not only enhances the safety and efficacy of regenerative therapies but also effectively circumvents the ethical issues associated with traditional stem cell sources. For instance, non-integrating reprogramming methods using small molecules can safely induce iPSCs without genetic modification. Similarly, decellularized extracellular matrix hydrogels (dECMHs) provide a supportive scaffold for MSCs, improving their viability and therapeutic efficacy. These innovations highlight the potential of synthetic materials in advancing tissue engineering while addressing ethical concerns.

**Fig. 7:**
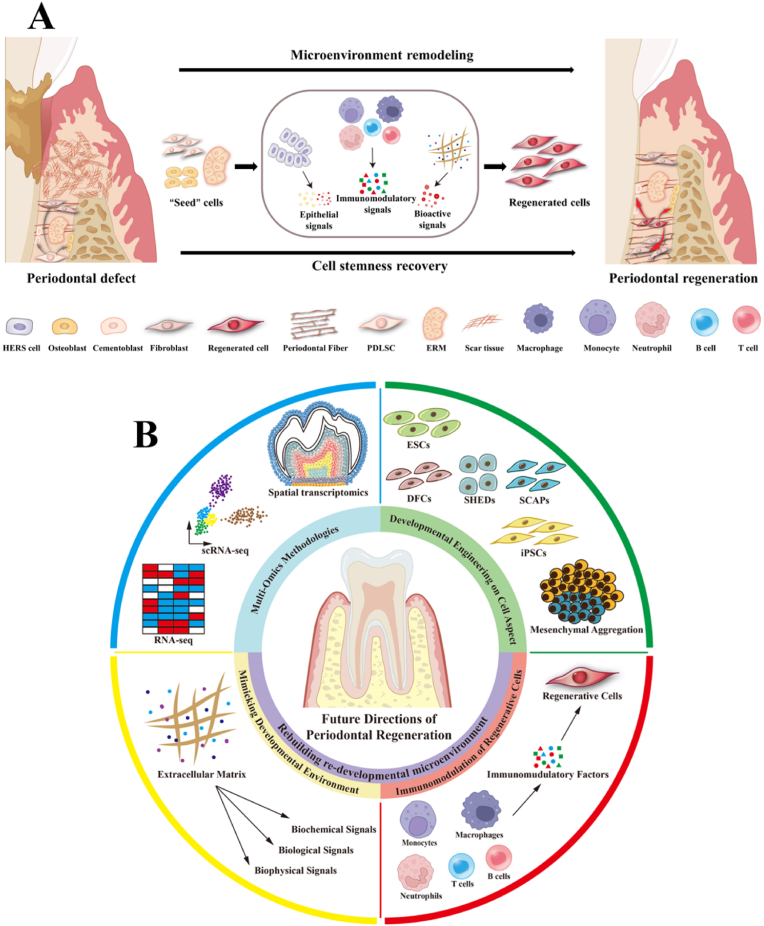
**The schematic figure for periodontal developmental engineering strategies and future directions.** (A) Strategies: developmental engineering highlights the importance of replicate developmental events, including developmental cells and developmental microenvironment. By monitoring the developmental events, it is expected to achieve periodontal complex regeneration. The developmental engineering holds great potential as a viable and ultimate approach for achieving periodontal complex regeneration. (B) Future directions: multi-omics methodologies include single-cell technology, spatial transcriptome sequencing and spatial proteomics could characterize the periodontal development and regeneration process to identify the vital cell types and genes. Application of cells such as ESCs, DFCs, SHEDs and SCAPs harvested from developmental process or inducing re-developmental state of cells like iPSCs and mesenchymal aggregation are great manner to perform developmental engineering on cell aspect. Immunomodulation of regenerative cells serves as a pivotal strategy to bring the periodontal repair process closer to the developmental process, facilitating the ultimate regeneration of the periodontal complex. Application of extracellular matrix of developmental tissues or cells is a great way to mimic the developmental environment to deliver biochemical, biological and biophysical signals to the regenerative area.

Thirdly, the consideration of clinical translation and industrial-scale production. Developmental engineering techniques, such as the use of advanced biomaterials, stem cells, and sophisticated bioprinting technologies, are resource-intensive and costly. These high costs can limit the accessibility and scalability of these strategies for widespread clinical application. However, our previous research identified hyaluronic acid (HA) as a unique component of the ECM in cranial osteo-organogenesis, where it was utilized as a novel engineered material for creating an “Osteo-organogenesis niche”. This approach restored immune surveillance and synergistically regulated stem cells to achieve re-osteo-organogenesis in cranial defects of rats [140]. HA, well-recognized for its extensive research and high biocompatibility, has been widely utilized in regenerative materials. Its synthesis is straightforward and cost-efficient, making it a promising candidate for clinical applications. Reassessing its role through the lens of developmental engineering offers novel insights and represents a promising avenue for the development of periodontal regeneration materials grounded in developmental engineering principles. The integration of advanced technologies such as 3D bioprinting, organoid culture, and nanomedicine further enhances the capabilities of developmental engineering. These technologies enable the creation of complex tissue structures that closely resemble natural tissues. Meanwhile, the thorough development of existing materials, such as hyaluronic acid (HA), which we have proven to possess developmental-inductive properties, contributes to cost savings and reduces ethical risks. Additionally, the application of serum-free media ensures the efficient expansion and maintenance of stem cell potency, while scalable industrial processes ensure the viability of these strategies for large-scale production. Continued research and collaboration across diverse scientific disciplines are essential to overcome current challenges and unlock the full potential of developmental engineering in regenerative medicine. While developmental engineering shows great promise, translating these strategies into clinical practice requires rigorous testing and validation to ensure safety and efficacy (Fig. 7).

## 5 Conclusions

**Fig. 8:**
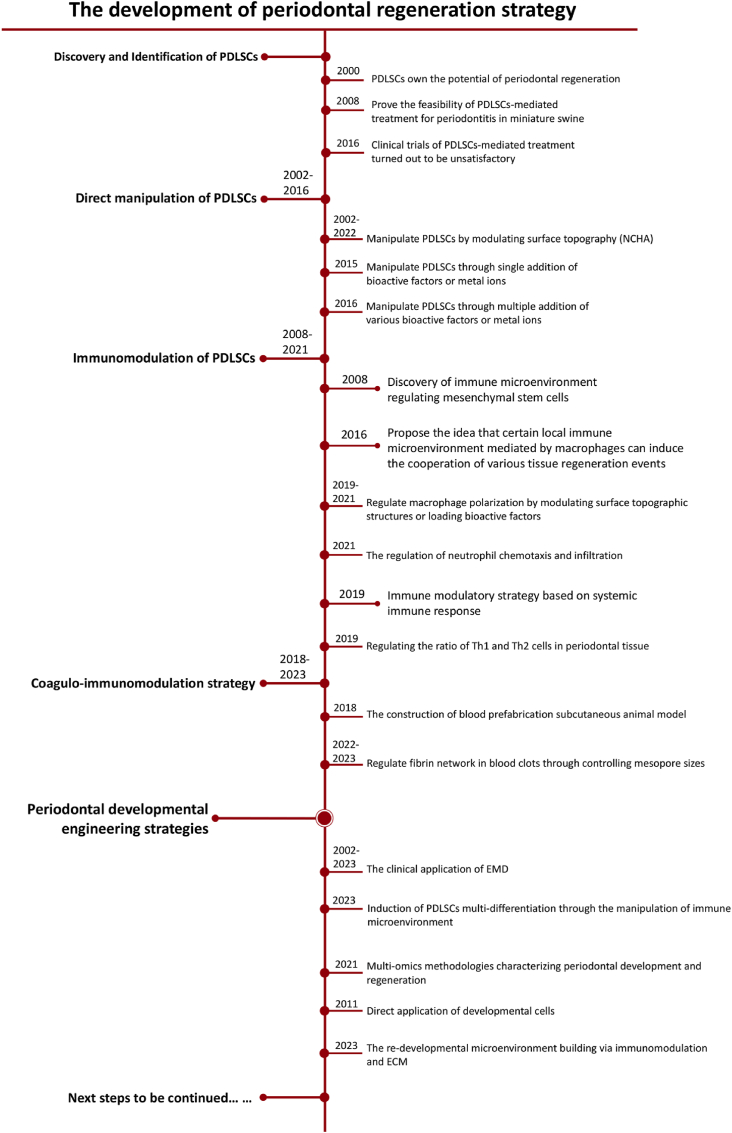
**The evolution of periodontal regeneration therapy.** The biological principle of periodontal regeneration has transformed from PDLSCs manipulation to periodontal developmental engineering. Based on PDLSCs manipulation, the periodontal regeneration therapy has evolved from direct manipulation, immunomodulation to coagulo-immunomodulation strategy. However, the functional activity of PDLSCs significantly declined with the development and maturation of periodontal tissue, resulting in periodontal regenerative disability. The next generation of developmental engineering strategies have been proposed and appreciated. Developmental engineering highlights the importance of replicating developmental events, including developmental cells and developmental microenvironment. The developmental engineering holds great potential as a viable and ultimate approach for achieving periodontal complex regeneration.

Periodontal complex regeneration remains a significant clinical challenge. As the understanding of periodontal complex regeneration gradually deepens, the corresponding regeneration strategies are constantly evolving. This review provides an overview of the strategic shifts in periodontal regeneration, emphasizing the transition from traditional approaches to more advanced developmental engineering strategies. It highlights the limitations of current methods and the need for a deeper understanding of the biological processes underlying periodontal development and regeneration. Future research should prioritize the application of multi-omics technologies to identify key cell types and microenvironmental factors involved in both processes. Additionally, the integration of advanced technologies such as 3D bioprinting and nanomedicine should be explored to create more accurate developmental microenvironments. The development of cost-effective and ethically sound materials, such as hyaluronic acid, offers a promising direction for clinical translation. By addressing these priorities, the field can move closer to achieving true periodontal complex regeneration (Fig. 8).

## CRediT authorship contribution statement

**Guanqi Liu:** Writing – original draft, Funding acquisition, Conceptualization. **Junlong Xue:** Writing – original draft, Visualization, Methodology. **Xuan Zhou:** Writing – original draft, Conceptualization. **Mixiao Gui:** Writing – original draft, Visualization, Methodology. **Ruidi Xia:** Writing – original draft. **Yanshu Zhang:** Visualization. **Yihua Cai:** Writing – original draft, Visualization. **Shuhua Li:** Writing – original draft. **Songtao Shi:** Writing – review & editing, Conceptualization. **Xueli Mao:** Conceptualization. **Zetao Chen:** Writing – review & editing, Funding acquisition, Conceptualization.

## Ethics approval and consent to participate

The animal and human subjects involved in this paper were performed in compliance with relevant laws and institutional guidelines. The consent was obtained for experimentation with human subjects.

## Declaration of competing interest

The authors declare that they have no known competing financial interests or personal relationships that could have appeared to influence the work reported in this paper.
